# Feasibility of delivering a digital multiple health behaviour intervention via a home-telemonitoring system: engagement, outcomes, and user experiences

**DOI:** 10.1186/s13104-026-07915-2

**Published:** 2026-07-07

**Authors:** Elizabeth S. Collier, Marie Löf, Preben Bendtsen, Joel Crawford, Marcus Bendtsen

**Affiliations:** 1 Department of Health, Medicine and Caring Sciences, Linköping University, Linköping, 581 83 Sweden; 2 Department of Food Research & Innovation, RISE Research Institutes of Sweden, Stockholm, Sweden; 3 Department of Medicine Huddinge, Karolinska Institute, Stockholm, Sweden

**Keywords:** Public health, Lifestyle, Dissemination method, Digital intervention, Behaviour change

## Abstract

**Objective:**

Finding novels ways to deliver digital health behaviour interventions could increase their reach and impact. The aim of this study was to evaluate the feasibility of using a home-telemonitoring system (offered to eligible patients by regional authorities in Sweden for symptom- and health marker monitoring) as a novel dissemination opportunity. The intervention has been previously found effective at changing health behaviours in a general online help-seeking population. In the present study, participants were not randomised: all had access to the intervention (pre-post analyses).

**Results:**

Among 416 patients offered the digital intervention, 12.0% (*n* = 50) signed up, 58.0% (*n* = 29) of whom completed the 16-week follow-up. After 16-weeks of intervention access, participants reported increased fruit and vegetable intake (median = + 95.4 g/day) and decreased candy consumption (median= -58.4 g/week), but little change in alcohol consumption or physical activity. Changes were non-linear throughout the study, with the largest changes observed at the beginning and after 8-week follow-up. Participants were generally satisfied with the support, though concerns of personal relevance, disproportionate personal responsibility, low content specificity, and frustration with repetitive reminders were noted. With intervention tailoring to the population and delivery system, home-telemonitoring systems could offer a way to increase proactive intervention reach beyond individuals already seeking help.

**Supplementary Information:**

The online version contains supplementary material available at 10.1186/s13104-026-07915-2.

## Introduction

Behavioural interventions, which can be delivered in person via primary healthcare or digitally, can help individuals lead healthier lifestyles by supporting favourable changes in alcohol consumption [1, 2], smoking [3, 4], diet e.g., fruit and vegetable (F&V) intake [5], and moderate-to-vigorous physical activity (MVPA) [6]. These interventions can target a single behaviour or several simultaneously, and typically work by leveraging behavioural change techniques (BCTs [7–9]) to engage psychological mechanisms of change e.g., increasing self-efficacy and practical know-how [10–12].

A digital multiple health intervention, “Coach”, targeting alcohol, smoking, diet, and MVPA was recently trialled in Sweden [13, 14]. The core of the intervention involved sending a text message to participants on Sunday afternoons, prompting them to self-monitor and reflect on their behaviours over the past week, and engage with materials designed to support behaviour change [14]. The trial found that there were benefits of having access to the materials, in particular, increases in F&V consumption were observed [13]. The “Coach” trial invited participants from the general population seeking help online to try to change their behaviour. However, to increase potential impact, novel ways of reaching into the community to deliver digital interventions should be investigated. In Sweden, some regional authorities offer eligible patients the use of a home-telemonitoring system allowing patient self-report about health measures e.g., blood pressure, weight, and general health status as measured by various relevant questionnaires. Patients use the system to report their values to healthcare staff via a web-portal where they could receive written or face-to face video feedback. The aim of the present study was to investigate the feasibility of offering the “Coach” intervention using such a home-telemonitoring system as the first point of contact.

## Methods

### Ethics

The Swedish Ethical Review Authority approved this study on 2023-07-04 (Dnr 2023-03091-01). The study was conducted in accordance with the Declaration of Helsinki and data was collected, stored, and processed in accordance with the General Data Protection Regulation (EU) 2016/679. Digital informed consent for study participation was obtained from all participants. This study was part of the MoBILE research program [15]. Clinical trial number: not applicable.

### Settings and participants

**Fig. 1:**
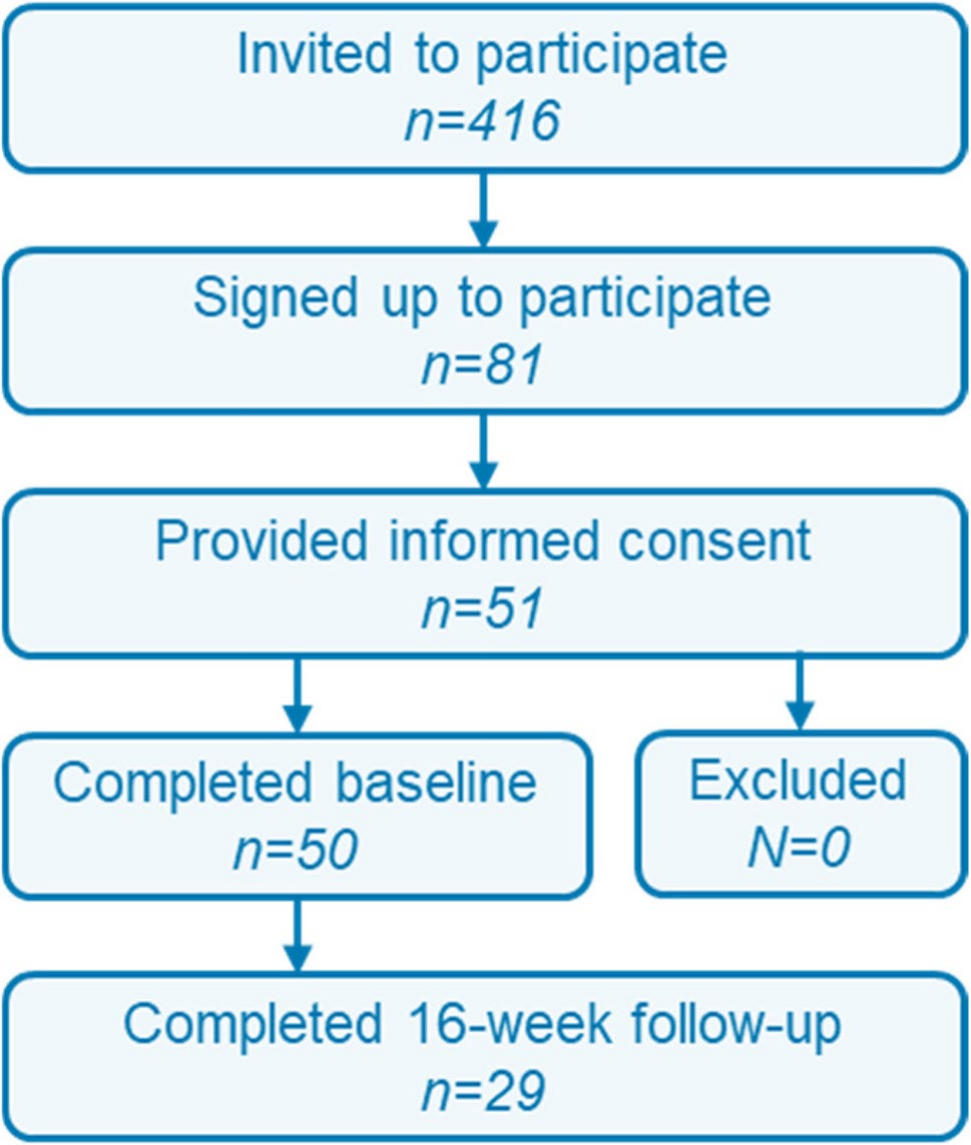
Flow diagram describing the sample size at various stages of the study

Participating patients were registered at one of five primary healthcare clinics in County Östergötland in Sweden. The two major patient groups were asthma and hypertension patients, followed by patients with heart failure. A message was sent through the home-telemonitoring system with instructions to send an SMS to a dedicated phone number to sign up for the study (two reminders sent, one week apart). The intervention was subsequently delivered via their mobile phone. Four-hundred-sixteen patients were invited to participate: 81 signed up, 51 consented, 0 were excluded, and 50 completed the baseline questionnaire (pick-up rate = 12.0%; Fig. 1). Twenty-nine participants completed the 16-week follow-up questionnaire and were largely representative of the recruited cohort (Table 1).

**Table 1:** Baseline characteristics of participants who were recruited (*n*=50) and those who completed the follow-up questionnaire at 16-weeks (*n*=29)

		Recruited (*n*=50)	Complete at 16-weeks (*n*=29)
Demographics	Age, mean years (SD)	62.2 (9.0)	62.7 (10.3)
	Sex, *n* (%)		
	Women	32 (64.0%)	20 (68.0%)
	Men	18 (36.0%)	9 (31.0%)
	Education, *n* (%)		
	Primary	9 (18.0%)	6 (21.0%)
	Secondary	21 (42.0%)	11 (28.0%)
	Tertiary	20 (40.0%)	12 (41.0%)
		BMI, n (%)	
	Normal (18.5-24.9)	11 (22.0%)	7 (24.0%)
	Overweight (25-29.9)	19 (38.0%)	7 (24.0%)
	Obese (30+)	20 (40.0%)	15 (52.0%)
Alcohol	Total weekly alcohol consumption (standard drinks), mean (SD)	3.5 (4.8)	3.5 (5.5)
	Weekly heavy episodic drinking, mean (SD)	0.9 (1.4)	0.8 (1.3)
Smoking	Number of smokers, *n* (%)	1 (2.0%)	1 (3.4%)
	Number of cigarettes last week, mean (SD)	0.02 (0.1)	0.03 (0.2)
Physical activity	MVPA (minutes), mean (SD)	258.3 (256.6)	265.3 (271.6)
Diet	Daily F&V consumption (g), mean (SD)	179.0 (103.7)	178.3 (109.7)
	Weekly sugar-sweetened beverages (330ml cans), mean (SD)	0.4 (1.0)	0.3 (0.5)
	Weekly candy (g), mean (SD)	175.0 (186.6)	193.1 (211.2)
Mediators ^a^	Importance, median score (quartiles)	8.0 (6.0; 9.0)	7.0 (6.0; 9.0)
	Confidence, median score (quartiles)	6.0 (5.0; 8.0)	6.0 (5.0; 8.0)
	Know-how, median score (quartiles)	8.0 (6.0; 9.0)	7.0 (5.0; 8.0)

^a^ Single items measured using a 1 (low) – 10 (high) ordered category scale; Importance reflects behavioural beliefs in which the motivation to change is influenced by the belief of how valuable a desired outcome [13]. Know-how, or having knowledge of how to change, reflects control beliefs, where individuals feel they have the requisite knowledge or skills to achieve an outcome. Confidence i.e., self-efficacy, is another facet of control beliefs, in which people perceived themselves as having agency to perform the given behaviour.

### Intervention

**Fig. 2:**
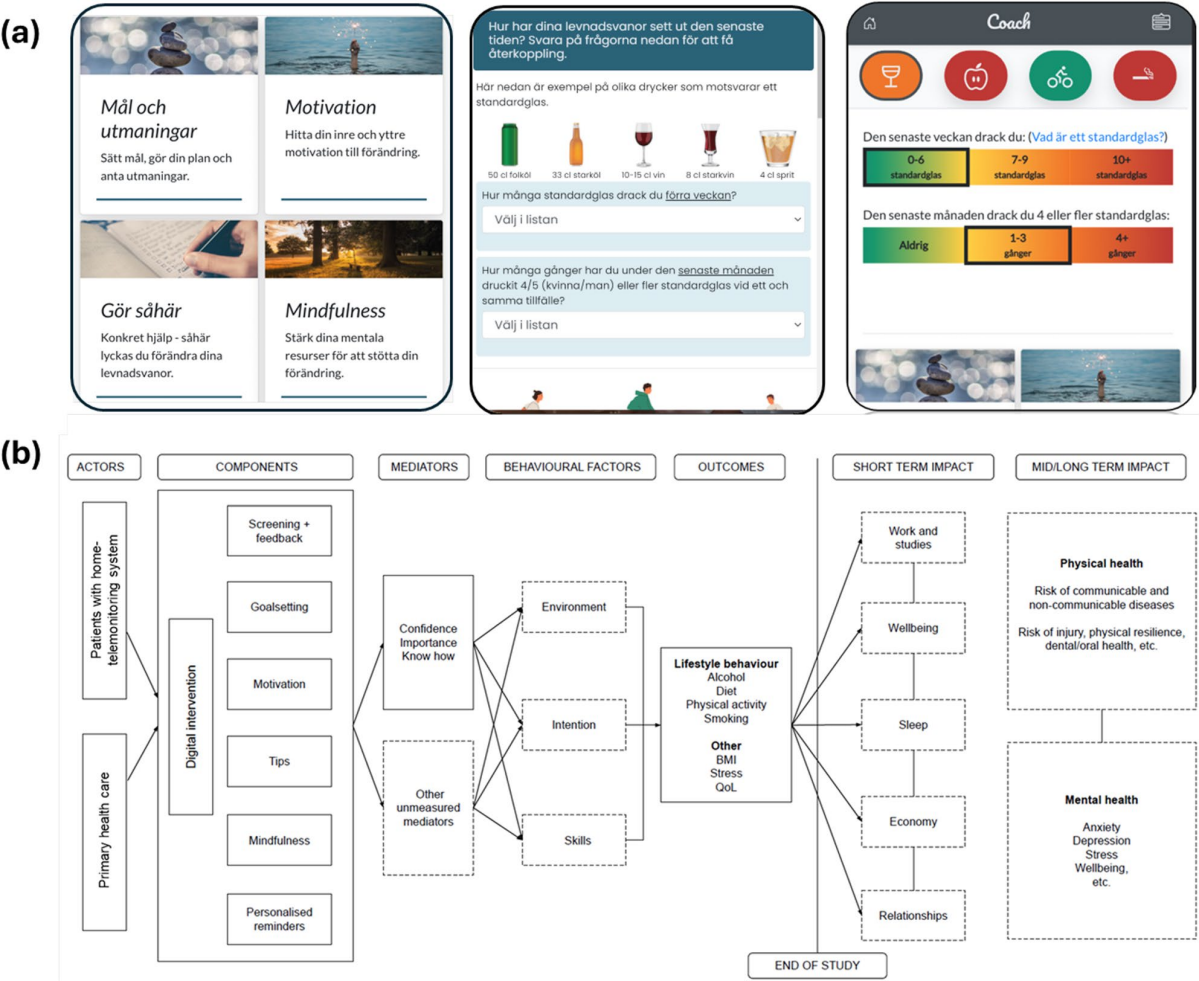
**a** Screenshots from the “Coach” digital intervention, showing the main menu as well as screening and feedback against national guidelines (left to right). **b** Logic diagram describing the reasoning behind the intervention, including actors, components, mediators, behavioural factors, outcomes and potential short-, mid- and long-term impact

Participants were not randomised, i.e., all participants had access to the full intervention. No inclusion/exclusion criteria were defined. The intervention, designed to modify participants’ motivation, intentions, and skills, contained a toolbox of six components (Screening/feedback, Goalsetting/planning, Motivation, Skills/know-How, Mindfulness, and Self-Authored Prompts) that participants accessed via their mobile phone and could navigate as they wished. Four health behaviours (alcohol, smoking, MVPA, and diet) were targeted. Screenshots of the intervention are shown in Fig. 2a and its underlying logic is illustrated in Fig. 2b).

Screening/feedback offered participants weekly behavioural screening, with feedback showing how their current behaviours aligned with national guidelines. Goalsetting/planning allowed participants to use a preset format targeting action and coping plans to set goals/plans for future behaviour and how they would reward success (“My goal is to ___. I will reach this by ___. If it gets difficult, I will do/say to myself that___. When I have reached my goal, I will give myself ___”). Participants could also take on challenges for the coming week, e.g., a daily 15 minute walk, or author their own challenges. Self-authored prompts offered participants to freely write messages to themselves (no guidance provided). Crawford et al. [13] and Supplementary Table S1 describe the components in detail.

### Outcomes

#### Health behaviours

The following health behaviours were measured at baseline, 8-week, and 16-week follow-up:

- Alcohol: Weekly alcohol consumption (TWC; standard glasses − 12 g pure alcohol); monthly frequency of heavy episodic drinking (HED; frequency consumed 4/5 (female/male) or more standard drinks of alcohol on a single occasion).
- MVPA: Weekly moderate-to-vigorous physical activity (minutes).
- Diet: Average daily consumption of fruit and vegetables (F&V; 100 g portions, converted to grams); weekly consumption of candy and snacks (50 g portions, converted to grams); weekly consumption of sugar-sweetened beverages (33 cl portions).
- Smoking: Four-week point prevalence of smoking abstinence.

#### Engagement

The number of participants engaging with, and the qualia of the produced content, was evaluated for screening/feedback, goalsetting/planning, and self-authored prompts modules.

#### User experiences

A follow-up questionnaire concerning how well the support fit participant needs (one qualitative and five quantitative questions, used previously to evaluate experiences of a digital alcohol intervention [16]) was completed at 16-week follow-up. The questions and responses are provided in the results section.

### Data analysis

Self-reported health behaviours at 16-weeks were compared to baseline values, adjusting for gender and age, using linear regression with Bayesian inference in *rstan* (Student’s *t* priors with 3 degrees of freedom and a scale of 2.5 for all coefficients; normal priors, µ = 0, σ = 10, for the intercept). The median of the posterior distribution was used as a point estimate of association, alongside 95% compatibility intervals (CoI) and probability of association (PoA).

Natural cubic spline modelling, using weeks 1, 8, and 16 as knots (representing study beginning, middle and end), was used to evaluate the screening data for patterns of change during the trial. Smoking data was not analysed (*n* = 1).

The number of goals, challenges, and prompts authored was counted. The percentage of challenges targeting each health behaviour was calculated. The content of the goals was evaluated independently by two authors (E.S.C., J.C.) through goal-setting theory [16] to assess valence (approach/avoidance/monitoring) and reward type (intrinsic/extrinsic/social/mixed). Goals targeting multiple behaviours were evaluated separately for each behaviour. Response frequency to each option on the quantitative experience questions was calculated, and content analysis was conducted on the open-ended participant experience data by a single author (E.S.C.).

## Results

### Change in health behaviours

At 16-week follow-up (Table 2), candy consumption decreased (median= -58.4 g/week; CoI= -134.4–3.4 g; PoA = 93.3%) and F&V increased (median = 95.4 g/day; CoI = 29.0–163.6 g; PoA = 99.7%), where intake of vegetables (+ 56.2 g/day) increased slightly more than fruit (+ 41.8 g/day). Weaker evidence existed for decreased TWC (median=-0.8, CoI=-2.5–0.8; PoA = 83.9%). Other behaviours did not markedly change. Women increased F&V and decreased candy more than men; these changes were also stronger among younger than older individuals. Older individuals reduced TWC slightly more than younger.

**Table 2:** Median of the posterior distribution over the effect of intervention, gender, and age on health behaviours at 16-week follow-up

		Median of the posterior distribution (95% CoI) *Probability of association in direction of the median*		
Health Behaviour		16-weeks vs. Baseline	Gender	Age
Alcohol	TWC: Total Weekly Consumption (standard glasses)	-0.8 (-2.5; 0.8) *83.9%*	-0.3 (-2.3; 1.5) *65.5%*	0.1 (0.0; 0.2) *99.1%*
	HED: Heavy Drinking Episodes (weekly)	0.2 (-0.5; 1.0) *73.7%*	0.1 (-0.8; 1.0) *55.1%*	0.0 (-0.04; 0.04) *51.4%*
Physical Activity	Weekly MVPA (minutes)	0.2 (-7.7; 8.8) *51.5%*	0.1 (-7.9; 9.4) *51.4%*	0.7 (-1.0; 2.7) *78.4%*
	Weekly moderate (minutes)	-0.1 (--6.4; 7.1) *51.2%*	0.1 (-8.7; 8.1) *51.5%*	0.3 (-1.4; 2.1) *64.7%*
	Weekly vigorous (minutes)	0.6 (-6.2; 11.1) *59.0%*	0.2 (-6.1; 8.2) *53.4%*	0.4 (-0.2; 1.1) *91.0%*
Diet	Daily F&V intake (g)	95.4 (29.0; 163.6) *99.7%*	52.5 (-27.9; 132.5) *89.8%*	-2.5 (6.2; 1.3) *90.4%*
	Daily fruit intake (g)	41.8 (4.7; 78.6) *98.5%*	29.3 (-19.1; 77.5) *87.5%*	-1.3 (-3.4; 1.0) *87.5%*
	Daily vegetable intake (g)	56.2 (21.9; 91.7) *99.9%*	21.8 (-22.2; 67.8) *83.8%*	-1.3 (-3.3; 1.0) *89.4%*
	Weekly candy (g)	-58.4 (-134.4; 3.4) *93.3%*	-70.0 (-23.9; 167.9) *92.8%*	-2.8 (-7.3; 1.6) *90.1%*
	Weekly sugar-sweetened beverages (units)	0.0 (-0.27; 0.26) *50.3%*	0.16 (-0.17; 0.51) *84.4%*	0.0 (-0.02; 0.01) *52.3%*

**Fig. 3:**
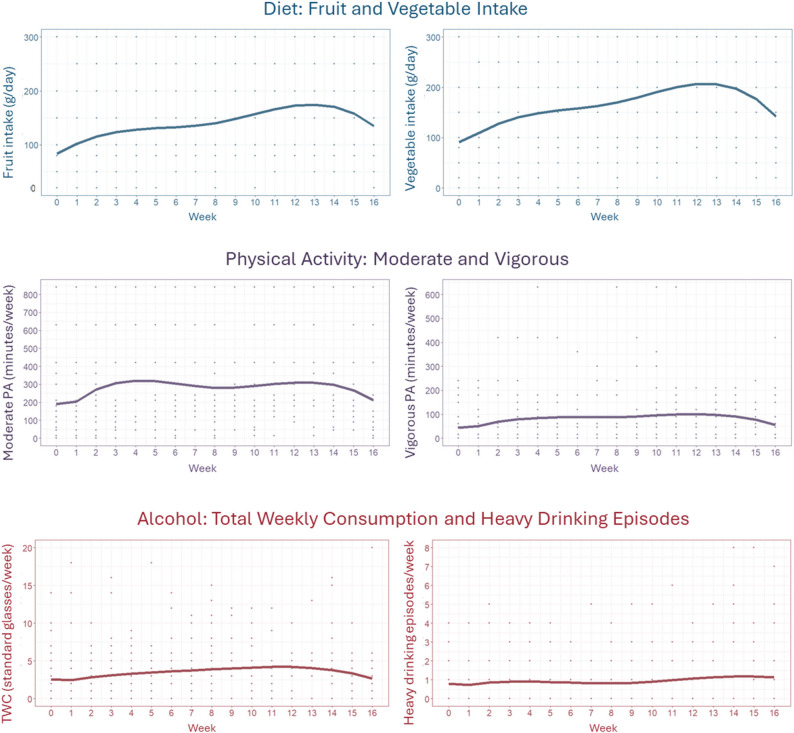
Cubic spline regression models showing change in health behaviours across the study duration (dots show individual data points)

Change was non-linear throughout the study (Fig. 3). Favourable changes in all behaviours were more marked at study start and after follow-up at 8-weeks, followed by a plateau which often transitioned into a downward trend. The observed increase in F&V was not linear week-by-week. Increases in physical activity were detected but not sustained between start and 8-weeks, and again between 8-weeks and 16-weeks. TWC showed a slight, gradual increase until 8-week follow-up after which it tapered back towards, and eventually dipped below, baseline. These associations should be treated with caution as the number of participants self-screening decreased across the trial duration: from *n* = 45 to *n* = 34 between weeks 0–1 and reaching a low of *n* = 13 during weeks 12–13 (Supplementals Figure S1). Screening increased in tandem with follow-up at 8- and 16-weeks (both *n* = 29).

### Engagement

Across 16-weeks, 21 participants took on 115 challenges; 22 authored 42 goals; and one authored a single prompt. Although most participants selected or authored 1–3 challenges/goals, one participant authored 45 challenges and nine goals.

Challenges targeted diet (49.6%), MVPA (24.3%), alcohol (21.7%), smoking (1.7%), and custom behaviours (2.6%, e.g., breathing exercises). Goals targeted MVPA (51.1%), diet (26.6%), alcohol (13.3%), and custom behaviours (8.8%, e.g., housework). The majority of goals were approach orientated (73.3%, e.g., eating more F&V), followed by avoidance (24.4%, e.g., abstaining from drinking), and mixed (2.2%, e.g., increasing F&V and decreasing candy), with none categorised as monitoring. Rewards were mostly intrinsic (55.6%, e.g., praise or time to relax), followed by extrinsic (35.6%, e.g., buying plants, clothes) or mixed (8.9%, e.g., coffee in the hammock, reflecting both rest and obtaining an external item), with none categorised as social. Goal development and reward exploration within individuals was observed, e.g., one participant increased their physical activity target from 40 to 60 min between goals, and some participants varied between intrinsic and extrinsic rewards.

### User experiences

Most participants reported that they received at least some support that at least somewhat met their needs, believed the support would be somewhat valuable to others, and would recommend the support to a friend looking to change their health behaviours (Table 3). The length of time individuals would like to keep using the support for was evenly split across response options.

**Table 3:** Questions and responses from the follow-up user experience questionnaire

Question	Response	Frequency (*n*, %)
(1) How well do you feel the support that you received met your needs?	I feel that I didn’t receive any support at all	3 (10.7%)
	I feel that I received some support, but it didn’t meet my needs	8 (28.6%)
	I feel that I received some support, and it somewhat met my needs	15 (53.6%)
	I feel that I received the support that I needed	2 (7.1%)
(2) You have responded that you feel like you did not receive support, or that the support you did received did not meet your needs. What did you do instead? [answered only by individuals responding (a) or (b) to question 1, *n* = 10]	I decided to find different support to change my lifestyle behaviours	3 (30.0%)
	I decided not to change my lifestyle behaviours	3 (30.0%)
	Other	4 (40.0%)
(3) Leave a comment describing your needs and how the support you received either met or did not meet those needs (open-ended question)	Open-ended response	26 (90.0%)
(4) To what extent do you believe that the support you received could help others who wish to improve their lifestyle behaviours?	1 – Not Very Helpful	2 (7.4%)
	2	1 (3.7%)
	3	6 (22.2%)
	4	12 (44.4%)
	5 – Very helpful	6 (22.2%)
(5) Would you recommend the support that you received to a friend who has expressed wanting to change their lifestyle behaviours?	Yes	17 (63.0%)
	No	2 (7.4%)
	Don’t know	8 (29.6%)
(6) If you were to continue using the support, how much longer would you like to have access to it?	1–2 months	1 (3.7%)
	3–6 months	5 (18.5%)
	6 + months	4 (14.8%)
	No more	6 (22.2%)
	Don’t know	11 (40.7%)

Two participants did not answer question 3–5, and one participant did not answer question 1

Reported positive aspects included increased motivation to eat well and exercise, receiving consistent and frequent reminders, gaining insight into their health behaviours, and increased reflection on their habits, promoting self-recognition of problem areas and subsequently taking action. Participants who did not feel the support suited them reported: already perceiving themselves as having healthy lifestyle behaviours (e.g., not drinking/smoking, exercising daily), disliking the expectation to take sole responsibility to change, finding the content surface-level/non-specific, and finding the reminders repetitive. Given initial contact via the home-telemonitoring system, one participant expected to see meaningful connections between lifestyle factors and the system’s purpose i.e., monitoring their medications and blood pressure. This fed into a broader feeling that the support was impersonal, with some mentioning specific medical conditions that limited content relevance and capability to follow the advice. Suggested improvements included providing recipes, meal-preparation ideas, and providing longer-term support.

## Discussion

The present study investigated the possibility of using a home-telemonitoring system as a new approach to disseminate a digital multiple health behaviour intervention. A pick-up rate of 12.0% was observed, which would likely increase if intervention access was not contingent on being part of a study and signing up via SMS. Considering the proactive approach and the novel way of reaching participants, this dissemination method appears at face-value to be feasible.

Though not directly comparable given differences in setting and population, it is notable that the results for diet align with those from the main “Coach” trial [13]. Relatively stronger changes in F&V and candy intake were observed here, which can likely be explained by regression to the mean being present in the current study. Further, participants in the current study rated changing their health behaviours as less important than those in the main “Coach” trial (8/10 vs. 10/10), which reflects the help-seeking behaviour of the main trial participants compared to the proactive recruitment done in this study. This also suggests that this dissemination method expanded reach beyond those actively seeking help as individuals less invested in behavioural change still partook, though the sample was still self-selecting.

Most participants felt like they received at least some support: positive aspects (increased motivation, self-reflection etc.) were noted, and interactive components e.g., goals and challenges, were appealing to at least some participants. The variety in goal valence and reward type, within and across individuals, suggests that individuals felt encouraged to explore the concept of goalsetting. Nonetheless, not all participants were satisfied. Similar concerns to those voiced here (personal relevance, disproportionate personal responsibility, low specificity, repetitive reminders) have been previously reported among digital intervention participants [17]. It is important that interventions feel personally relevant, as this can increase effectiveness [18]. Personal relevance may be especially pertinent for patient populations presenting with a range of diagnoses and conditions, since general advice may not apply and different barriers (physical, psychological, and structural) could exist. Delivery context also affects expectations. As mentioned by a participant here, first contact via the home-telemonitoring system created the expectation that connections would be made between the physiological/medical and lifestyle data they logged. This would require both tailoring intervention content to patient populations and having it dynamically interact with data logged in the home-telemonitoring system.

On balance, the results support that with adaptation to the delivery system and population, home-telemonitoring systems could offer a way to provide digital health behaviour interventions to the community. This could increase their impact by expanding reach beyond individuals already seeking help, to those who could benefit but have not yet actively planned to change their lifestyle.

### Limitations

The lack of randomisation or a control group limits inference to associations only. The sample size is small and self-selecting, though this was likely due to the previously mentioned barriers to entry (e.g., study participation). Nonetheless, future work with larger samples would be beneficial. The sample did not optimally reflect the target population: ~48.0% of home-telemonitoring system users are male, versus 36.0% of study participants. Measures were also self-report.

## Supplementary Information

Supplementary Material 1.

## Data Availability

The datasets used and/or analysed during the current study are available from the corresponding author on reasonable request.
