# Supplementary material for: Feasibility of delivering a digital multiple health behaviour intervention via a home-telemonitoring system: engagement, outcomes, and user experiences
Source: BMC Res Notes. 2026 Jul 7;19:279. doi: 10.1186/s13104-026-07915-2 (PMC13339708; doi:10.1186/s13104-026-07915-2)
Supplement: Supplementary file 1 — Supplementary Material 1. [file 13104_2026_7915_MOESM1_ESM.pdf]

Supplementary Materials

Delivering a digital multiple health behaviour intervention via a home-telemonitoring system: feasibility, outcomes, engagement, and experiences

Elizabeth S. Collier*[1,2] , Marie Löf[3], Preben Bendtsen[1,], Joel Crawford[1] & Marcus Bendtsen[1]

[1] Linköping University, Department of Health, Medicine and Caring Sciences, Sweden.

[2] RISE Research Institutes of Sweden, Department of Food Research & Innovation, Sweden.

[3] Karolinska Institute, Department of Medicine Huddinge, Sweden.

Table S1: Description of the six components in the parent trial, "Coach"

Component	Description	Present/absent	Behavioural Change Technique(s) leveraged[§]
Screening and Feedback	Every Sunday afternoon, participants will receive a text message with a hyperlink which takes them to a questionnaire regarding their current health behaviours. Once complete, feedback on their current behaviour is given in relation to national guidelines. Thereafter users are given access to the rest of the components (depending on allocation).	When absent participants were not shown the questionnaire but instead only national guidelines without personal feedback.	Discrepancy between current behaviour and goal (BCT 1.6), Feedback on behaviour (BCT 2.2), Self-monitoring of behaviour (BCT 2.3) and Social comparison (BCT 6.2)
Goalsetting and Planning	This component let participants set a goal for their future behaviour and plan for what to do when they struggle and succeed. Participants can also accept challenges for the coming week, for example, to walk for 15min each day, or to not drink any alcohol this week. Self-composed challenges are also available. Reminders are sent via texts to participants about their goals and challenges throughout the week.	When absent, this component was not visible.	Goal-setting behaviour (BCT 1.1), Problem solving (BCT 1.2), Action planning (BCT 1.4), Prompts/cues (BCT 7.1), Behaviour practice/rehearsal (BCT 8.1), Behaviour substitution (BCT 8.2), Habit formation (BCT 8.3), Graded tasks (BCT 8.7)
Motivation	This component contains information and tools to increase participants' motivation for change. This includes information on negative health consequences, costs induced from certain behaviours and reflective tasks. If participants choose, they can also activate motivational text messages which are sent to them throughout the week.	When absent, this component was not visible, and text messages were not available.	Information about health consequences (BCT 5.1), Credible source (BCT 9.1), Pros and cons (BCT 9.2), Comparative imagining of future outcomes (BCT 9.3)
Skills and Know-how	Concrete tips on how to initiate and maintain change in everyday life is offered in this component. This includes giving participants strategies they can use to say no to alcoholic beverages at parties, how to increase the nutritional value of their breakfast, etc. If participants choose, they can also activate text messages with tips sent to them throughout the week.	When absent, this component was not visible, and text messages were not available.	Social support unspecified (BCT 3.1), Instructions on how to perform a behaviour (BCT 4.1), Self-incentive (BCT 10.7), and Self-reward (BCT 10.9)
Mindfulness	This component aims to increase users' awareness of their own lived experience and strengthen their capacity for non-reactive, compassionate and less stressful way of being in the world. Mindfulness exercises are offered to participants, including guided meditations.	When absent, this component was not visible, and guided meditations not available.	N/A
Self-composed prompts	Participants are given the opportunity to compose messages and have them sent to themselves throughout the week (on days and times of their own choosing). A participant may for instance write a message to themselves reminding them to eat two fruits each day, to not drink anything on Wednesdays, or to go for a walk with a friend.	When absent, this component was not visible.	Problem solving (BCT 1.2)

[§] *The Coach trial was designed prior to the publication of the BCT Ontology in 2024 and so the BCT Taxonomy was used.*

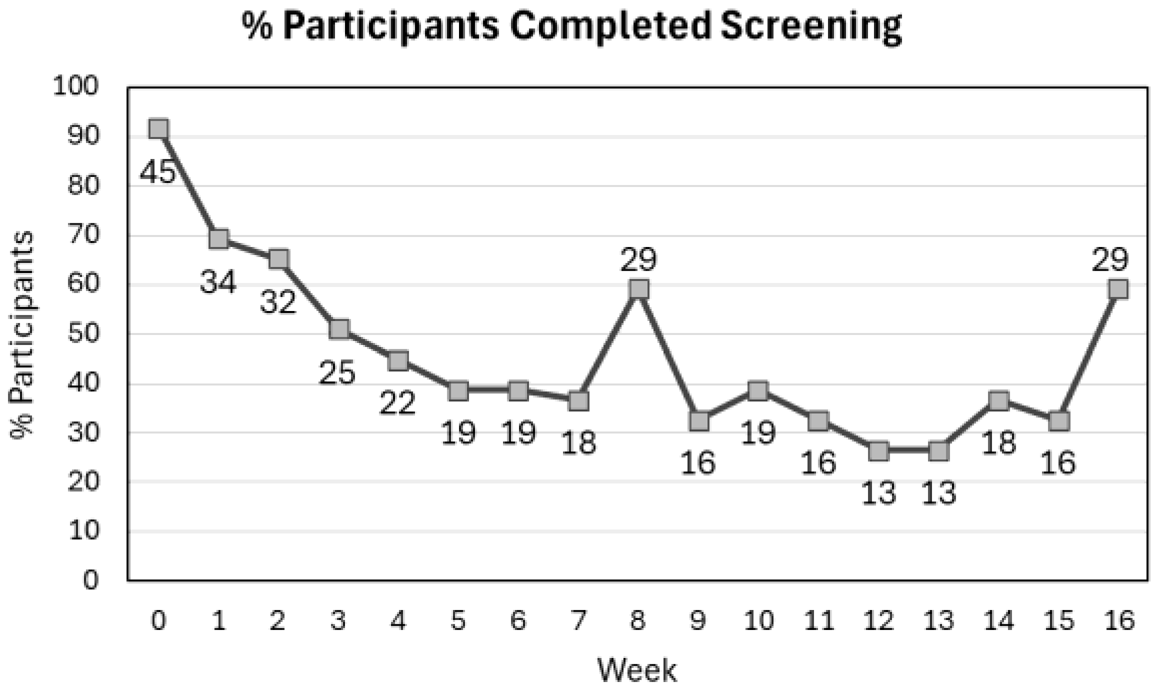

Figure S1: Number and percentage of participants screening their health behaviours during each week of the study
